# Epidemiological and Clinical Patterns of Newly Diagnosed Hepatocellular Carcinoma in Brazil: the Need for Liver Disease Screening Programs Based on Real-World Data

**DOI:** 10.1007/s12029-020-00508-7

**Published:** 2020-09-12

**Authors:** Gustavo dos Santos Fernandes, Daniel Campos, Andre Ballalai, Rodrigo Palhares, Mario R. A. da Silva, Daniel M. F. Palhares, Ben-Hur F. Neto, Fabio M. do R. Barros, Roberto de A. Gil, Aline Chagas, Flair José Carrilho

**Affiliations:** 1 Centro de Oncologia, Hospital Sírio-Libanês, SGAS 613/614, Conjunto E, Lote 95, Brasilia, DF CEP 70200730 Brazil; 2 Sociedade Brasileira de Oncologia Clínica, Sao Paulo, SP Brazil; 3 IQVIA Brasil, Sao Paulo, SP Brazil; 4 Bayer Brasil, Sao Paulo, SP Brazil; 5 Hospital de Clínicas de Porto Alegre, Universidade Federal do Rio Grande do Sul, Porto Alegre, RS Brazil; 6 Consultant Surgeon at Diagnósticos da América SA (DASA) e Associação Brasileira de Linfoma e Leucemia (ABRALE), Sao Paulo, SP Brazil; 7 Real Hospital Português de Beneficência, Recife, PE Brazil; 8 Serviço de Oncologia Clínica do INCA e Oncoclínica Centro de Tratamento Oncológico, Rio de Janeiro, RJ Brazil; 9 Hospital das Clínicas da Faculdade de Medicina da USP e Instituto do Câncer do Estado de São Paulo (ICESP), Sao Paulo, SP Brazil

**Keywords:** Hepatocellular carcinoma, Epidemiology, Public health system, Brazil

## Abstract

**Purpose:**

Describe sociodemographic and clinical characteristics of patients with hepatocellular carcinoma (HCC) and establish their history in the Brazilian public health system.

**Methods:**

Retrospective observational study was conducted using the database from the Department of Informatics of the Unified Health System (DataSUS). Patients with at least one claim of HCC between July/2011 and June/2016 were included. A record linkage methodology was performed to obtain longitudinal data across different databases. Demographic and clinical data were evaluated, including the time elapsed between diagnosis of HCC risk-factors and the cancer development. Data was analyzed using descriptive statistics.

**Results:**

A total of 28,822 HCC cases were identified between July/2011 and June/2016. Mean age was 59.7 years (SD = 14.7), and most patients were men (55.9%). The highest relative number of HCC cases was detected in the south of Brazil (> 20 cases/100,000 inhabitants). About 86.5% of the patients had diagnosis of HCC without previous liver diseases. Only 8% had diagnosis of chronic viral hepatitis and 3.5% cirrhosis. About 76% were diagnosed at an advanced stage, and only 11% of the patients had early stage HCC. Approximately 58% of patients with previous underlying liver diseases were diagnosed at early stages, compared with only 24% of patients without prior record of underlying diseases.

**Conclusion:**

The diagnosis of HCC in the Brazilian public health is usually made in patients with no previous diagnosis of liver disease and in advanced stages, when no curative treatment is available and survival rates are low. Public health policies are key for the screening and monitoring liver disease and, consequently, HCC.

## Introduction

Liver cancer is one of the most common types of cancers and the third cause of cancer-related mortality in the world [1, 2]. It was estimated that in 2018, there were more than 840,000 new cases of liver cancer and almost 782,000 deaths worldwide, with an increasing trend in the number of cases [1, 2]. To aggravate the scenario, it presents a poor prognosis, leading to a high socioeconomic burden [3].

Hepatocellular carcinoma (HCC) is the most common type of liver cancer, accounting for 85–90% of cases [1]. Varied risk factors, such as infection by hepatitis B and C (HBV and HCV, respectively), among others, may cause chronic liver inflammation, which in turn causes abnormal hepatocyte proliferation and the development of HCC. Regardless of the etiology, liver cirrhosis is present in 80% of HCC cases [4, 5]. For this reason, it is key to detect and manage the risk factor involved in HCC development.

Despite being a global public health issue, the incidence of HCC varies according to the specificities of each geographic region. For instance, in sub-Saharan Africa and eastern Asia, HCC cases are closely related to the incidence of HBV infection, while in Europe, North America, and Japan, HCV is the main risk factor involved [1, 6]. In Latin America, there is a heterogeneous scenario for HCC; most cases are related to HCV and alcoholic liver disease; however, there are countries where the main cause is HBV infection [7, 8]. Cirrhosis is the main risk factor for the development of HCC. In Brazil, most cases are associated with chronic hepatitis B and C infection and alcohol consumption [9]. In recent years, non-alcoholic fatty liver disease (NAFLD) has emerged as an important cause of chronic liver disease, cirrhosis, and HCC, and due to the absence of effective surveillance strategies, the presentation stage is often advanced [9]. In Brazil, liver cancer is responsible for 0.7% of tumors in the country, with a 5-year prevalence estimated in 5.2 cases per 100,000 inhabitants and an incidence of 2.7 new cases per 100,000 inhabitants [10]. However, due to its severity, it has high mortality rates with only 25–30% of patients stage III and 10–20% of patients stage IV disease living 5 years [11].

Although recent advances in therapeutic approaches to HCC have improved patient prognosis, it is still associated with low cure rates and long-term survival, especially due to its advanced stage at diagnosis [12]. Early diagnosis of HCC allows curative procedures and is associated with increase survival and reduced mortality [12]. However, data on HCC prevalence and risk factors in Brazil are still scarce. In this context, the study aims to analyze the sociodemographic and clinical profile of HCC patients in the public Brazilian healthcare system (SUS), to generate evidence to support decision making process and public health policies.

## Methods

This retrospective observational database study evaluated sociodemographic and clinical characteristics of patients with HCC in the Brazilian public healthcare system. The study was conducted using data available in DataSUS, the largest health database in Brazil. DataSUS is the information system from the Ministry of Health, and it is composed by different databases. In order to achieve the study objectives, it was analyzed the databases of both outpatient system (*Sistema de Informações Ambulatoriais*, SIA) and the inpatient system (*Sistema de Informações Hospitalares*, SIH) between July 2011 and June 2016. Based on the Brazilian regulation, this study was not submitted to the ethics committee as the data is public available and it was reported in an aggregated form.

### Participants

This study aimed to cover the largest possible number of patients with HCC available in the databases. For this reason, we included all the patients that presented at least one medical record for HCC in the DataSUS databases from July/2011 to June/2016. The HCC diagnosis was identified based on the International Classification of Disease (ICD-10), with the following ICD codes: C22.0 (liver cell carcinoma), C22.9 (unspecified malignant liver neoplasia), and C22.7 (other specified liver carcinomas). The last two ICD codes were also considered to avoid underestimation on the number of patients with HCC due to inaccuracy at the initial diagnosis. No exclusion criteria were used.

### Study Design

For this study, the outpatient (SIA) and inpatient (SIH) databases were extracted from DataSUS (www.datasus.gov.br). SIA contains (1) demographic data, such as date of birth, gender, and city, among others; (2) procedures related to the treatment of the disease; and (3) patient unique identifier code. In the other hand, SIH contains (1) demographic data and (2) hospitalization information, such as procedures, time, and type of hospitalization. Once SIH does not contain the unique identifier code from SIA, the record linkage methodology was used to integrate both databases to provide individual and longitudinal information of HCC patients in SUS.

The record linkage methodology consists in deterministic and probabilistic connections of the two databases. To connect the data, parameters such as ZIP code, city, date of birth, age, gender, race, nationality, and ICD-10 were used in 18 steps with different combinations. Then, it defines an anonymized primary key for each patient, which allowed the combination of both databases.

The data obtained from both databases was validated by combining several variables. The results are considered valid only if no inconsistencies of date of birth and gender are detected for the same patient [12]. In addition, patients who are not found in one of the databases receive new codes and are kept in the final database. This prevents the loss of the records in one of the databases. The final database provides a longitudinal patient information with sociodemographic characteristics, health-related procedures, and underlying diseases.

### Outcomes and Variables

As SIA and SIH are basically reimbursement related databases, they do not have all clinical information available. Age, gender, and geographic distribution was analyzed at first medical record reported. The diagnosis date was determined as the date of first mention of HCC in medical records. In addition, it also evaluated the presence of risk factors based on the following ICD-10 codes: B18.0 and B18.1, for HBV; B18.2 for HCV; and K70.2, K70.3, K71.7, K74.0, K74.1, K74.2, K74.3, K74.4, K74.5, and K74.6, for cirrhosis. The time from risk factors to HCC development was also described for the patients based on a longitudinal analysis.

One important variable that was not available in the database was the staging of HCC. Therefore, the procedures performed for HCC, in correlation with their indications by national and international protocols available at the time, were used to determine the staging [13, 14].

Patients were considered at an early stage when curative procedures such as hepatectomy and liver transplantation were performed. For intermediate stages, the procedures of arterial chemoembolization (TACE), except if the patient did TACE to control the tumor and underwent to curative surgery, radiotherapy, and ethanol percutaneous injection, if tumor size was larger than 2 cm, were used to classify the patients. Advanced stage patients were classified using the presence of palliative systemic treatment at diagnosis. The terminal stage was defined by patients with HCC registration who were declared dead in the database or by patients who did not perform any of the mentioned procedures, with at least 6 months of registration in the databases and more than 1 year without further registration.

### Statistical Analysis

As this is a descriptive exploratory database study, no sample size was calculated. No formal hypothesis was tested in the study; the data was analyzed using only descriptive statistics. The results were described using the mean (SD), median (interquartile range (IQR)) for continuous variables, and numbers and frequencies (%) for categorical variables.

Patient’s age was calculated based on the date of birth and date of diagnosis (first appearance of HCC), and it was categorized in intervals of 10 years. Regarding the geographic distribution, the analysis was performed using the city of the patient and the Brazilian population was obtained from the Brazilian Institute of Geography and Statistics (*Instituto Brasileiro de Geografia e Estatística*, IBGE). The number of patients was adjusted by the population provided by IBGE. Regarding the time from risk factor to the diagnosis of HCC, it was calculated using the date of risk factor first appearance and date HCC diagnosis. Then, the average of this difference was calculated. All analyzes were performed using R software.

## Results

### Patients Characteristics

**Fig. 1:**
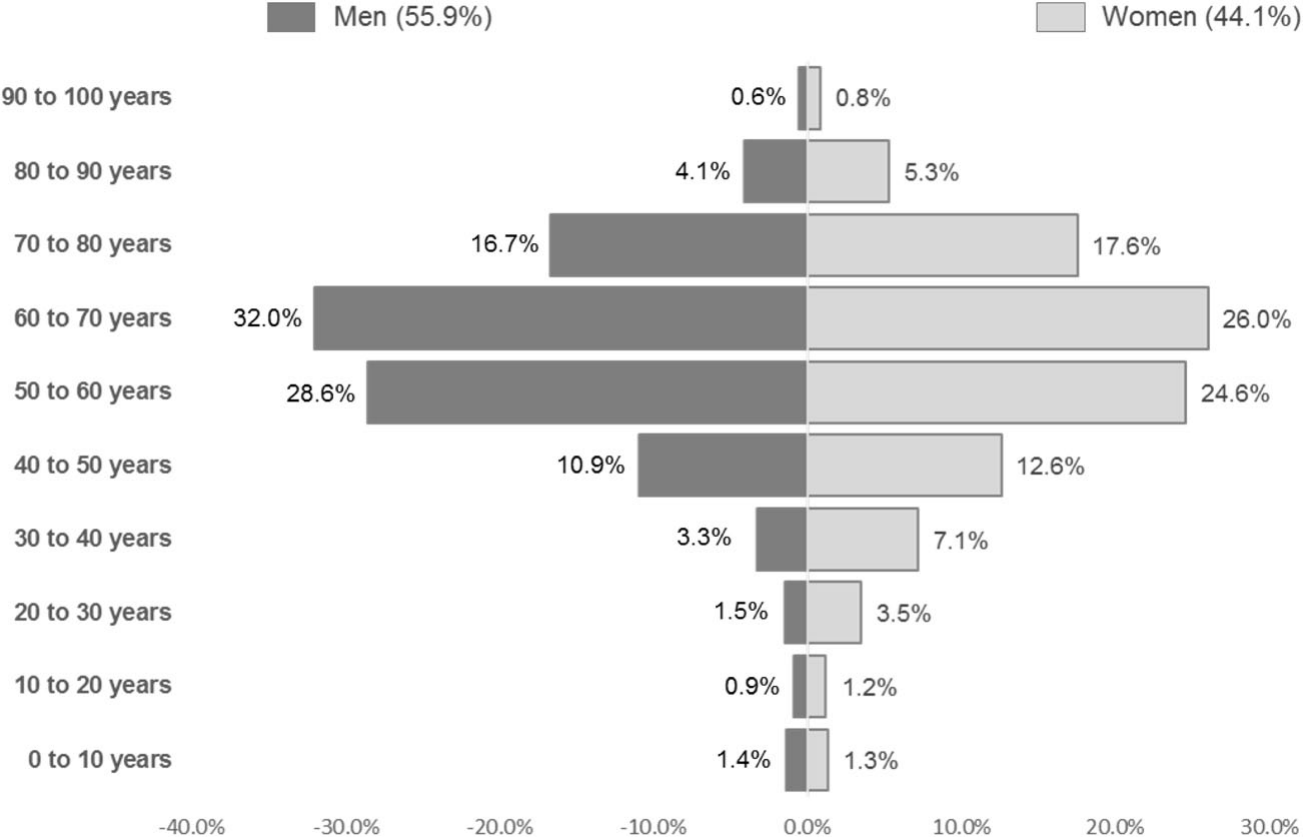
Distribution of population with HCC treated by SUS by gender and age

A total of 28,822 patients HCC patients were identified in the databases between July/2011 and June/2016. Most patients with HCC were men (55.9%), and the mean age was 59.7 (SD 14.7) years old. The complete distribution of patients’ gender by age category is described in Fig. 1.

**Fig. 2:**
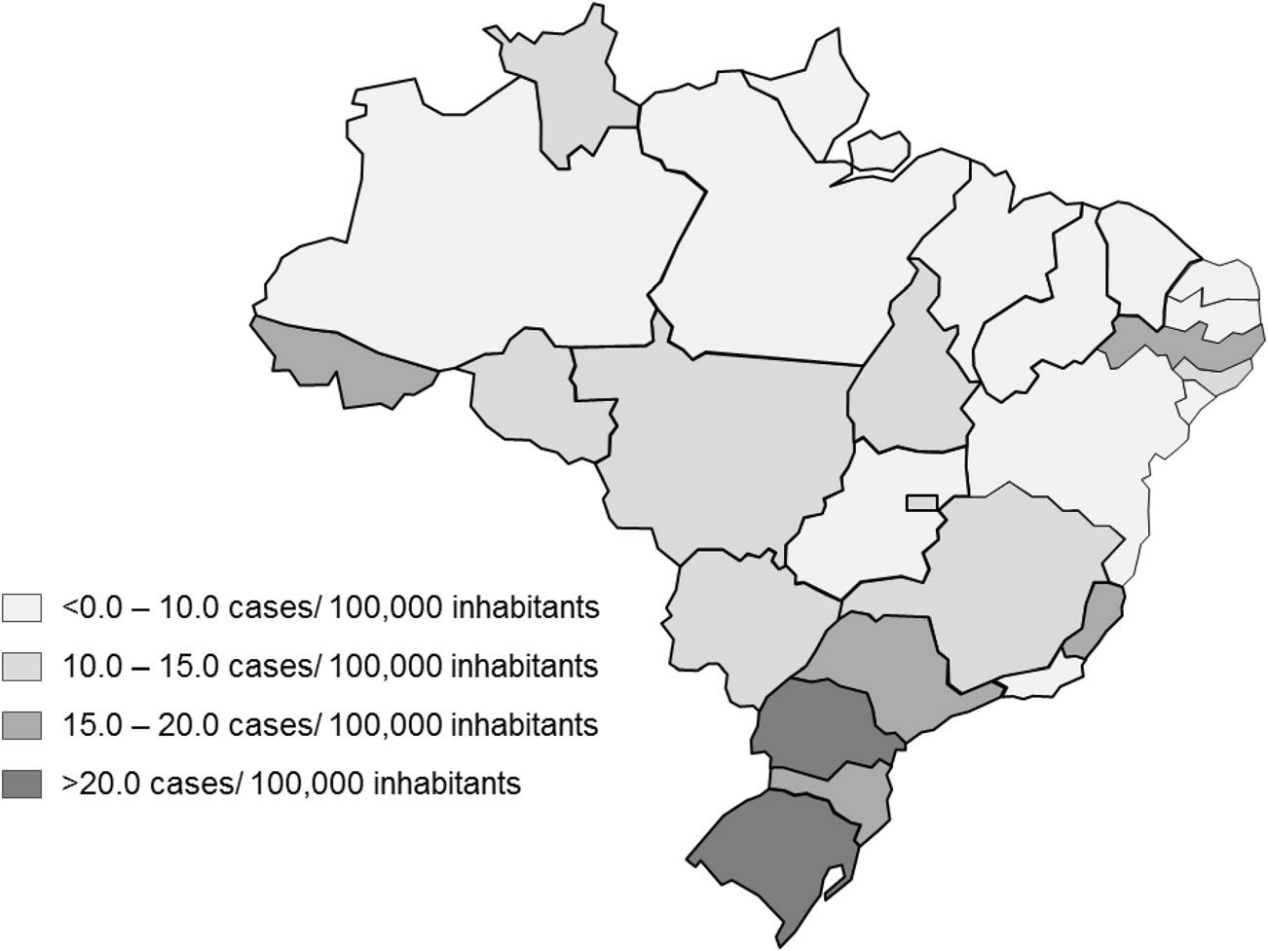
Concentration of HCC cases in SUS by state

São Paulo state presented the highest absolute number of patients identified. However, after the data was adjusted by the population, the highest relative number of HCC cases was found in the south of (states of Rio Grande do Sul and Paraná) with more than 20 cases/100,000 inhabitants. The patient distribution by state is shown on Fig. 2.

### Clinical Characteristics

Regarding the presence of risk factors, 86.5% of patients were diagnosed with HCC without previous record of viral hepatitis or cirrhosis, while 8.0% were previously diagnosed with HBV or HCV, 3.5% with cirrhosis, and 1.8% with both diseases before the diagnosis of HCC.

**Fig. 3:**
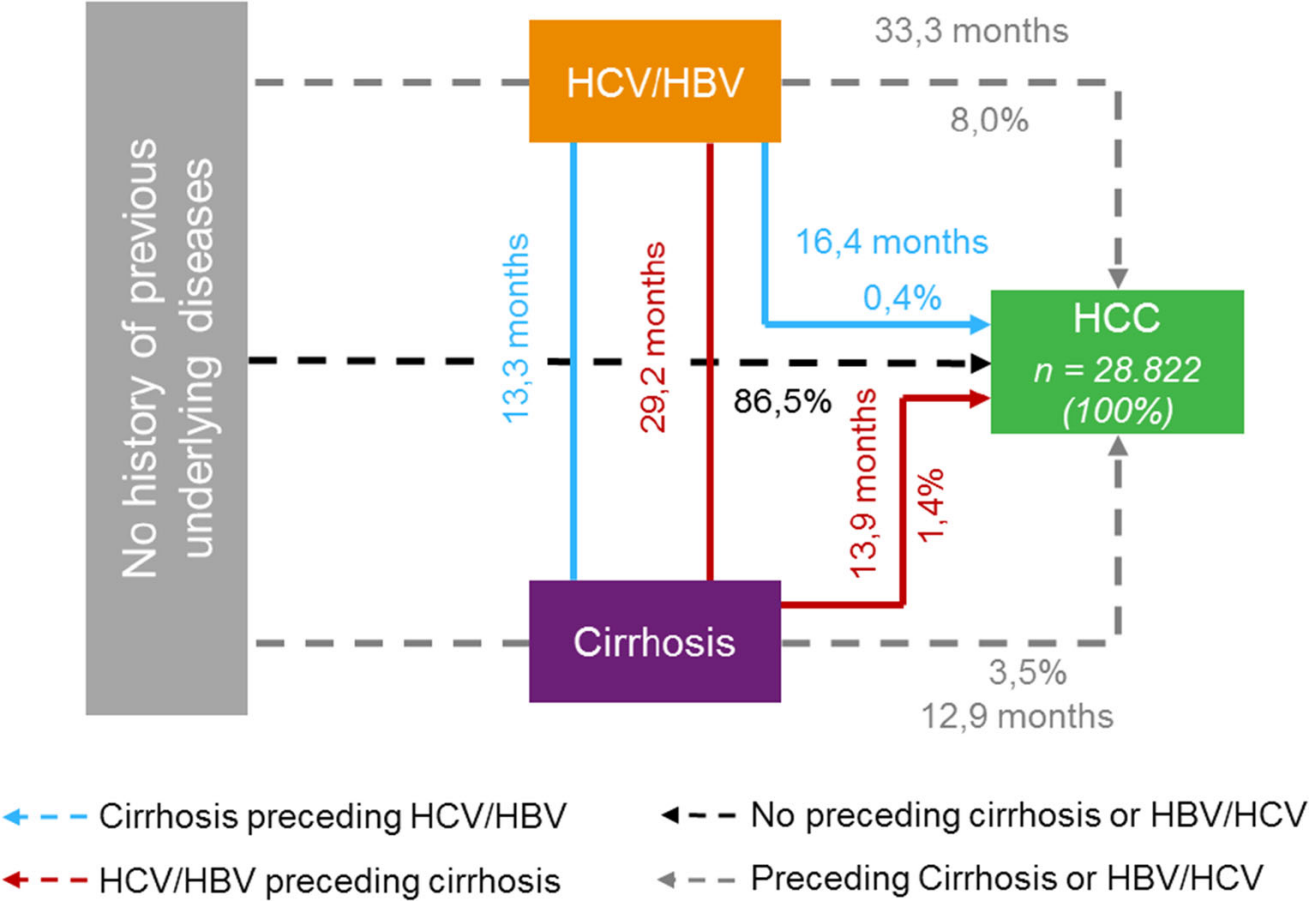
Patient´s journey in SUS and progression time from underlying diseases

Of those patients who presented baseline disease records prior to HCC, the time from the underlying disease and HCC was calculated. The time varied from 12.9 months in patients with cirrhosis to HCC and 33.3 months in patients with HBV and HCV to HCC. The proportion of patients with each risk factors and the time from it to HCC diagnosis is presented in Figure 3.

**Fig. 4:**
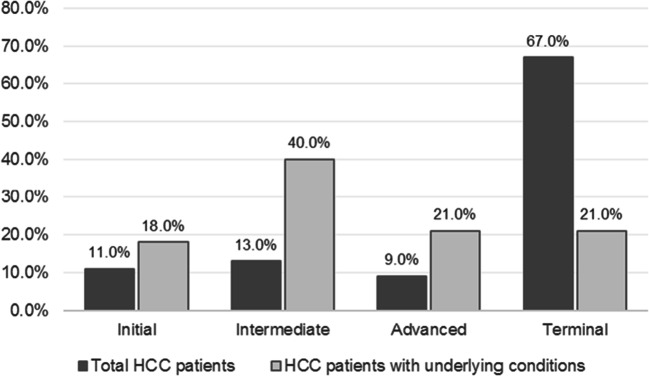
Staging of patients in the total population and in the population previously diagnosed with underlying liver disease

Figure 4 shows the staging analysis according to the presence of risk factors. Early-stage patients represent 11% of the sample, intermediate-stage 13%, advanced-stage on chemotherapy 9%, and the other 67% are on terminal stage, under palliative care.

## Discussion

This observational database study evaluated the sociodemographic and clinical characteristics of the patients with HCC identified in the DataSUS, the largest health database in Brazil, and evaluated the largest sample of HCC patients in the country. In addition, it was possible to calculate the adjusted number of cases by state and the time from the presence of risk factors to the diagnosis of HCC. The study showed that most patients were men and the mean age at diagnosis was 59.7 years. In the literature, there are evidences corroborating these findings. It is known that HCC occurs more frequently among men than in women; in some countries, the difference between incidence rates reaches 3:1 [12, 13]. In general, men not only have higher chances to have risk factors—such as HBV infection, alcohol, and tobacco consumption, for instance—but also present gender-related factors that contribute with the development of the liver cancer [1]. HCC is more prevalent in older adults and elder, with the number of cases increasing from the age of 50 and reaching its peak around 60–70 years, as described in the literature [1, 13, 14]. In addition, similar results that corroborate our findings have been demonstrated in previous studies conducted in Brazil [15–17].

Moreover, the study also evaluated the number of cases adjusted by the population of each state, based on IBGE estimates. São Paulo state had the highest absolute number of cases identified in the database. However, the south of Brazil had the highest relative number of cases (> 20 cases/100,000 inhabitants). This may be related to the fact that these states present a higher proportion of old adults and elder, which are the most prevalent age of development of HCC. In addition, a study showed that HCV infection was more prevalent in Southern states and in urban areas, which may also contribute to the higher number of cases of HCC in these states [18]. Regardless of etiology, this data may be an indicative for further studies and public health policies in these states.

It was observed that 86.5% of HCC patients did not present previous record of underlying liver diseases, and only 5.3% of patients were previously diagnosed with cirrhosis. However, in the literature, different studies conducted in southeastern tertiary care centers have reported that approximately 90% of patients with HCC had cirrhosis as the underlying condition [9, 17, 18]. This difference between our results and the literature suggests that in Brazilian public health system, there is an enormous lack of diagnosis and monitoring of risk factors, such as HBV/HCV and cirrhosis, as HCC is often diagnosed without these underlying conditions and in more advanced stages. In any case, it is also important to highlight that the data entered in DataSUS should be part of the explanation for such discrepant numbers. First, while patients with HCC are diagnosed with an advanced form of the disease and require immediate treatment, the underlying diseases might not be the focus of therapy and their ICD-10s records may not be inserted in DataSUS system. Second, in some degree, may exist underreporting of risk factors that might impact the interpretation of the data by official organs such as the Ministry of Health and, consequently, limit the adoption of public health policies with focus on the prevention or treatment of underlying liver diseases.

When underlying liver diseases were present, HCC was diagnosed at earlier stages, as shown in Fig. 4. This result suggests that in some cases, the screening for liver disease is being performed and that it is key for the early detection of HCC, which will certainly influence the prognosis and costs involved in the disease management. For instance, a study showed that HCC patients with underlying HBV are hospitalized approximately five times a year, 8 to 12 days for each hospitalization [18, 19]. Therefore, the lack of risk factors’ monitoring causes an important socioeconomic impact.

The heterogeneity on patients screening and monitoring for liver diseases can be observed in the literature. A multicenter study showed that the 6-month follow-up time for screening of liver disease is not adequately followed, especially in the public system [20]. In another study conducted in a reference center in the southeast of Brazil, 35.7% of patients with HCC were diagnosed at an early stage, 23.4% at an intermediate stage, 31.6% in advanced stage, and 9.3% in terminal stage [20]. Based on our findings, it is possible to observe that in general, 24% of the HCC cases are diagnosed at initial and intermediate stages in the Brazilian scenario, but in reference centers occur the opposite, with 59.1% of the patients being diagnosed at the same stages [21]. In another reference center study in Brazil, in newly diagnosed HCC patients, it was found the following BCLC distribution: 25 (4.9%) were BCLC-0, 246 (47.4%) BCLC-A, 107 (20.6%) BCLC-B, 76 (14.6%) BCLC-C, and 65 (12.5%) BCLC-D [22]. This result shows that this unfavorable scenario can be changed by monitoring at-risk patients, as it is performed in specialized institutions. Therefore, it is necessary to have a standardization of the screening and monitoring of liver disease to prevent late stage HCC diagnosis.

In short, because HCC is a silent disease, like cirrhosis and viral hepatitis, the diagnosis occurs in advanced stages of the disease, when procedures are no longer curative, but are costly and only focused on increasing patient’s survival [23]. Therefore, early diagnosis of liver disease and screening leading to early HCC diagnosis are crucial for reducing the number of HCC deaths.

Another important topic to be addressed is the importance of health information systems for monitoring the quality of SUS and healthcare institutions through performance indicators. For instance, in the case of cancer, there are still inconsistencies and underreporting in SUS information systems. These inconsistencies between diagnosis and treatments used, incorrect codes and lack of treatment start date, make the analysis of these databases difficult [23]. In addition to this, the health system has difficulties in ensuring an adequate dynamic of the patient through the different stages of care. Currently, the referral and counter-referral system between the different levels of SUS care is fragmented, especially in the context of chronic diseases, such as cancer and liver disease [24]. On one hand, the primary and secondary care services are not prepared to receive the patient from tertiary care; on the other hand, tertiary care services are often overwhelmed with services that could be offered in primary and secondary care levels. This deficiency in the dynamic of the patient through the healthcare network needs to be addressed to ensure complete care of HCC patients [24].

This study presents important limitations. First, it was conducted using the information from DataSUS, which covers only patients from the public healthcare system, which is approximately 70% of the Brazilian population. Moreover, the databases used in the study were only the outpatient (SIA) and inpatient (SIH), meaning that only patients who were submitted to a procedure or hospitalization were included in the study. For these reasons, it is possible that some patients may be missing from the analysis. All the patients with C22.0, C22.9, and C22.7 were included, and the interpretation is that other primary tumors from the liver and biliary system may have been inadvertently included. However, based on epidemiological factors, we have estimated that the number of non-HCC tumors is very small. Another important limitation was the lack of clinical information. These are reimbursement-related databases; therefore, not all clinical information was available for analysis. However, this is an expected limitation of retrospective observational studies. The staging of HCC was determined based on treatment and clinical protocols, which is subject to misinterpretation. In addition, it is important to notice that while databases provide patient death information, there might be underreporting due to patients who died outside the hospital and whose records are not closed in the system. For these reasons, the study results should be interpreted carefully. Despite the above limitations, the use of these databases has the advantage of a broader analysis of the disease, with a larger number of patients, allowing the generation of relevant evidence for decision making when it comes to public policies.

## Conclusion

In Brazilian public health system, HCC is diagnosed at an advanced stage, when no curative treatment is available and survival rates are low. The development of strategies to improve the screening of patients with underlying liver disease, through the diagnosis, treatment, and adequate follow-up of patients with risk factors, is essential to increase the diagnosis of early stage HCC when curative treatments can be performed, increasing survival rates and improving patients’ quality of life. It is necessary to broaden the discussion of coordinated actions from the early stages of the disease where screening and diagnosis are key factors, as well as the integration of actions throughout the treatment journey, so that effective strategies to reduce mortality by liver cancer are identified and implemented in the future.

## Data Availability

Yes. The study was done in accordance with guidelines of Good Clinical Practice and the Declaration of Helsinki, and with applicable regulatory requirements.
